# Analytical Investigation of the Micro Groove Surface Topography by Micro-Milling

**DOI:** 10.3390/mi10090582

**Published:** 2019-08-31

**Authors:** Jinfeng Zhang, Chao Feng, Hao Wang, Yadong Gong

**Affiliations:** 1 School of Mechanical Engineering, Shandong University of Science and Technology, Qingdao 266590, China; 2 School of Mechanical Engineering and Automation, Northeastern University, Shenyang 110819, China

**Keywords:** micro-milling, surface roughness, surface topography, minimum chip thickness

## Abstract

Micro-milling is an emerging processing technology for machining micro- and high-precision three dimensional parts that require the use of various materials (with sizes ranging from tens of micrometers to a few millimeters) in the field of advanced manufacturing. Therefore, it can be applied to manufacture the micro parts, but new challenges are raised about parts with high surface quality. Herein, both surface formation and micro machined surface roughness models are studied, with the aim of solving complicated problems regarding the quality of surface finish when micro-milling metallic materials. From a theoretical point of view, the first model for surface formation processes considering the strain gradient plasticity theory was built in the area around the cutting edge, and the minimum uncut chip thickness equation was derived. The model accounts for the properties of the work material in tertiary and quaternary zones on the minimum chip thickness. A second model for micro machined surface roughness based on the relationship of kinematics between cutting process and cutter edge was also developed, which takes the influences of tool run out into account. Both proposed models were introduced to analyze the tendency of surface roughness for micro grooves. Both models were also used to justify experimental results. The results show that the developed surface roughness model could be useful in predicting both roughness parameters and trends as a function of cutting parameters.

## 1. Introduction

With the development of science and technology, micro structures and parts play more and more important roles in aerospace, biomedicine, power, energy, etc. Micro-scale milling has now become an emerging mechanical machining process, which can be used to produce complex 3D micro parts with a variety of materials. Moreover, it has a high material removal rate, low cost, and is easy to use [1]. However, the surface of machined material is not always ideal. Surface roughness refers to the micro geometric shape produced on the surface of material and has great influence on the performance of the parts. The surface quality can be improved by refining the existing processing experience for the parts in conventional milling. However, there are no mature processing rules for micro scale milling. In addition, the surface area-to-volume ratio becomes higher as the size of micro devices reduces anywhere from several microns to tens of microns, resulting in surface effects playing a more important role. Surface roughness is one of many criteria by which to evaluate the quality of a surface, i.e., whether the quality is great or not [2]. Therefore, it is especially necessary to better understand surface characteristics and the related mechanisms. 

The geometric models of tool and process conditions have been proven for predicting the surface generation. In micro scale milling, the minimum chip thickness is comparable in size to the edge radius, resulting in a large negative rake angle cutting [3]. The nature of the micro-deformation during ploughing or rubbing due to negative rake angle effect contributes significantly to increased surface roughness [4].

Yang et al. [5] investigated surface roughness using specific cutting energy. The specific cutting energy consumed on the up-milling side was smaller than that on the down-milling side. The variation of surface roughness on the centerline position of the micro-slots exhibited distinct characteristics from that in the macro-cutting. The extra cutting energy on the down-milling side was proven to be helpful for surface improvement of the groove bottom. Yuan et al. [6] pointed out that the diamond tool sharpness has a considerable influence on the machined surface integrity. In their study, surface roughness, micro-hardness, residual stress, and dislocation density of the machined surface layer were found to vary with the cutting edge radius. That is, the surface generated was strongly influenced by the minimum chip thickness. 

In micro-end milling, the feed rate is also roughly equal in magnitude to the edge radius of the tool, which is usually less than a few microns [4]. Liu et al. [7] indicated that the ratio of minimum chip thickness to tool edge radius, termed as the normalized minimum chip thickness, is determined by the material thermo-mechanical dependent properties. They found that the normalized minimum chip thickness increased as the cutting speed and the tool edge radius increased when machining carbon steels. This was attributed to the predominance of the thermal softening effect over the strain hardening effect for carbon steels. However, for micromachining AL6082-T6, the normalized minimum chip thickness was found to stay almost constant. Weule et al. [8] found that the surface roughness increased at low values of the feed rate relative to the tool edge radius. In addition, they also observed that the surface generated was strongly influenced by the microstructure for steel. Thus, the surface finish is not only determined by its own thermomechanical properties, such as yield strength and ductility, but also by the cutting conditions, such as cutting speed and tool edge radius. Wu et al. [9] developed a flow stress model based on strain gradient plasticity for the process of micro-cutting. It also related the flow stress to tool geometry and cutting conditions, which contain edge radius, rake angle, clearance angle, undeformed thickness, and cutting width. Sun et al. [10,11] presented an analytical model to predict the non-uniform surface. It found that the surface roughness in the center was lower than that on the sides. This was mainly attributed to the much larger ratio of stochastic surface roughness determined by the minimum chip thickness and the ploughing effect rather than the geometric surface roughness dominated by tool residual marks. Both feed rate and depth of cut have a pronounced influence on surface uniformity. While the cutting speed shows no apparent connection with surface uniformity, when the feed rate is lower than 1.6 µ m/flute, both the surface roughness and the surface uniformity decrease as the feed rate increases due to the increased length of the shearing interval in the center of grooves. The surface quality improves as the depth of cut increases. This attributes to the reduced ploughing effect. Jin and Altintas [12] presented a slip-line field model of the micro-cutting process with a round edge tool, which considers the stress variation in the material deformation region due to the tool edge radius effect including strain, strain-rate, and temperature effects.

In addition to the studies noted above, in conventional milling, there is abundant research for surface roughness; however, microscale surface quality is still in the exploratory stage in micro scale machining [13]. The experiment for micro-milling brass by Takacs [1] showed that surface roughness was not always good with smaller feed peer tooth. Wang [14] made the attempt at incorporating tool diameter, cutting depth, spindle speed, and feed rates into the mathematical model of surface roughness. He Ning [15] suggested that, provided the surface roughness becomes better with smaller cutting edge radius, the minimum cutting thickness could be reduced by changing the cutting tool and the material properties of the workpiece or the cutting condition. Gong [16] theoretically and experimentally investigated this notion with respect to aluminum alloy and titanium alloy. The results showed that the minimum cutting thickness effect was produced when the ratio of the feed per tooth to the radius of cutting edge was smaller than one. Moreover, the surface roughness (Ra) of the groove bottom was gradually reduced, and the numbers and the height difference of burrs distribution decreased with the increasing spindle speed and feed per tooth. Duan [17,18] proposed a theoretical method based on a least square vector algorithm for predicting surface roughness in high speed milling. According to the surface measurement result, it was found that workpiece surface roughness reflected most factors on the cutting process, including material hardness, cutting speed, cutting depth, and feed rates. Liu [19] studied and analyzed size effect phenomenon and found that, with the increase of feed per tooth, the surface roughness decreased first and then increased. It provided a theoretical basis for the reasonable selection of cutting parameters and the analysis of the actual processing of micro-milling size effects.

The height of the generated surface is mainly affected by the cutting tools run out during the process of micro-milling [20]. The process damping effect induced by the elastic recovery of work material and the effect of the tool-tip vibration are regarded as the prime influences on surface roughness [21]. Biondani et al. [22] developed a model considering cutting edge topography, material deformation, and cutting edge trajectory errors to achieve a reliable prediction of surface topography generation in ball end milling. To predict micro-milling surface roughness of Inconel 718, Lu et al. [23] combined the ideal cutting trajectory of micro-milling and the system dynamic response model, which considers minimum cutting thickness, multiple regenerative effects, and elastic recovery to obtain the actual cutting trajectory of a micro-milling cutter. The simulation model of micro-milling surface topography was established. The results showed that the built simulation model could predict surface topography. Yuan et al. [24] established an accurate surface roughness model that takes into account the combined effects of the run out, the minimum chip thickness, the trajectory of the tool tip, and the tool geometry for micro end-milling. It was validated by micro end-milling experiments. The results showed that the predicted and the experimental surface roughness showed similar variation trends and close amplitude levels. In addition, both minimum chip thickness and run out are dominant factors for surface generation in micro-end-milling.

From the discussions above, it is obvious that the surface processing system is far from established for micro-scale milling. There is a clear distinction between micro-milling and macro-milling due to the reduction of tool dimensions and machining variables. The change of milling parameters, especially spindle speed, feed rate, axial cutting depth, radial run out of cutting tool, structure of the material, etc., have a certain influence on the surface roughness. The existing theory and experience cannot be directly applied to all processes. In order to better grasp the law of the surface finish of the material to obtain high precision machining results and reduce the surface roughness of micro parts, more systematic and comprehensive experimental study is necessary. In this paper, the bottom surface of the micro channel was mainly experimentally studied, and theoretical analysis was carried out.

In response to the reviews above, the goal of this research is to provide a better understanding of the surface forming process. This paper presents a new model of micro machined surface formation and roughness using two-dimensional representation with a round edge tool element in Section 2. The indentation geometry of the rounded sections for the edge tool in the work material and the associated deformation region are analytically modeled by assuming the workpiece as a plastically isotropic material and considering the strain gradient plasticity in Section 2.1. The machined surface roughness is evaluated using the height of the residual area. The height is obtained as a function of cutting edge geometry, cutting processing parameters, and material properties in Section 2.2. The micro straight grooves corresponding to typical parts are machined by the developed three-axis linkage micro-milling machine tool in Section 3. The surface quality of micro grooves is investigated by using milling on different kinds of engineering materials in Section 4. This paper also presents an analysis of the cutting speed, the feed per tooth, the axial cutting depth, the structural materials, and the run out for the surface roughness over a wide range of engineering materials. Where possible, comparisons of the observed trends are made with those results in scientific literature for micro-scale cutting in Section 5. The paper is concluded in Section 6. The findings of this paper may potentially be applied to improve cutting and workpiece surface integrity and to provide a theoretical basis and technical guidance when micro-milling channels, slots, or cavities for manufacturing micro-parts.

## 2. Theoretical Model Analysis

### 2.1. Micro Surface Formation Models

**Figure 1:**
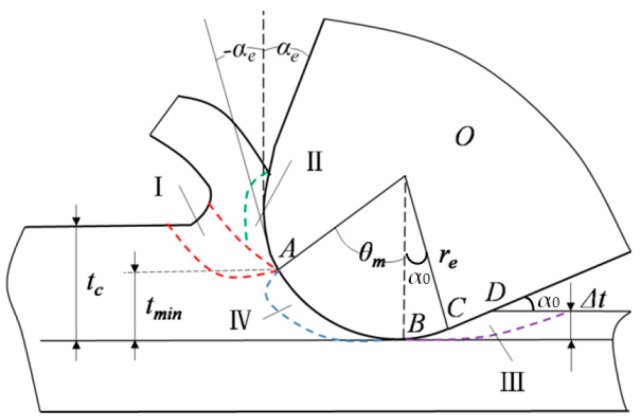
Micro machined surface formation process.

Shearing action at the primary and the secondary plastic zones gives rise to a shear-sliding deformation. The partial material is sheared to deform into a chip, and the other part is squeezed into the workpiece surface. When the uncut chip thickness is comparable to the radius of the cutting edge, the material removed is largely dependent on the area near the cutting edge. Thus, the influence of the material around the cutting edge must be considered in micro-milling. The model of the orthogonal micro-cutting process using two-dimensional representation with a round edge tool is presented. The material deformation region is divided into four zones: I, the primary zone, II, the secondary shear zone, III, the tertiary zone, and IV, the quaternary zone, as shown in Figure 1.

It is assumed that there are steady state cutting conditions in the model. The material separation stagnation point A is the intersection of the minimum cutting thickness and the arc of cutting edge. The material in regions III and IV under the stagnation point A is suppressed to form the finishing surface, while in regions I and II, at what was previously point A, plastic slipping deformation occurs. This is also known as the basic deformation zone or the main deformation zone in region I. It is also the main source of cutting force and cutting heat during the cutting process. When the chip is flowing out along the rake face into region II, it is further squeezed and rubbed. The direction of metal fibrosis near the rake face is parallel to the rake face, which is mainly frictional interface between tool and chip. The machined surface produces fibrosis and hardening in region III under the action of extrusion of partial cutting edge and flank face, which has a great influence on residual stress and flank wear. Region IV is defined in the vicinity of the tool‘s round edge. It is assumed to be dominated by the ploughing/rubbing and the scratching contact and affects the deformation of the chip and the production of the built-up edge.

The material of the cutting layer flows out and then becomes the chip based on the continuum plasticity theory regarding material deformation of the first and the second zones. The machined surface is formed when it is through the third and the fourth zone. As shown in Figure 1, the uncut chip thickness *t*_c_ cannot be completely removed, leaving a thin layer due to the existing tool edge radius *r*_e_. In other words, the uncut chip thickness *t*_c_ is bifurcated upward and downward when the cutting layer *t*_c_ passes through the point *A*, and the material previously at point *A* runs along the rake face and turns into a chip, and that under point *A* produces the elastic/plastic deformation and generates the finished surface. During the micro-end milling process, the thin layer has a magnitude of elastic recovery Δ*t* and leaves some material on the processed surface. Thus, the contact length of the flank face in the tertiary zone turns from *BC* to *BC + CD*, which increases the friction and the extrusion between the flank and the surface to be machined.

In micro-scale end milling operations, the depth of cut may be below 10 μm, and the anticipated surface roughness may only be a few nanometers. Note that the tool edge radius is comparable in size to the undeformed chip thickness. The rake angle of the tool is positive *α*_e_, which is not the actual rake angle participating in the processes due to the existence of the tool edge radius. As can be seen, the effective angle is negative −*α*_e_. *α*_0_ is the normal clearance angle. The stagnant angle *θ*_m_ is approximately equal to the friction angle *β* between the material and the rake face based on the minimum energy approach and the infinite shear strain approach [25], regardless of the other parameters involved in the process. The minimum chip thickness model can thus be determined from the previous geometrical relationship.

(1) $$\{\begin{array}{c} t_{\min}=r_{e}(1-\cos\theta_{m})=r_{e}(1-\cos\beta )\\ \alpha_{e}=\theta_{m}-\pi /2=\beta -\pi /2 \end{array}$$

A chip is not formed if the uncut chip thickness is set below the minimum chip thickness *t*_min_, and then the workpiece material is just plastic deformation and elastic recovery. This process is called the ploughing effect. It is dominant when the uncut chip thickness *t*_c_ is small enough, which makes the influence of elastic recovery of the machined surface more obvious. Thus, the amount of elastic recovery ∆*t* must be taken into account during micro-scale milling [3,26] in tertiary and quaternary zones.

(2) $$\left\{ \begin{array}{ll} \Delta t=\frac{3\sigma_{s}r_{e}}{4E}\left[2\exp(\frac{H}{\sigma_{s}}-\frac{1}{2})-1\right] & t_{c}>t_{\min}\\ \Delta t=t_{c} & t_{c}\leq t_{\min} \end{array}\right.$$

As shown in Equation (2), when the uncut chip thickness *t_c_* is larger than the minimum thickness *t*_min_, ∆*t* is decided by the radius of cutting edge *r*_e_, tensile strength of workpiece *σ*_s_, elasticity modulus *E*, as well as the ratio between hardness of workpiece *H* and tensile strength of workpiece *σ*_s_. When the cutting thickness *t_c_* is smaller than the minimum thickness *t*_min_, the amount of elastic recovery is approximately equal to the uncut chip thickness *t_c_*. It is obvious from previous formulation that the elastic recovery ∆*t* is linear to the tool edge raduis *r*_e_ with respect to the same type of material and process effects, such as tensile strength, hardness, modulus, temperature, strain rate, and so on.

Most of the material deformation around the cutting edge is largely accumulated in the third and the fourth areas due to the uncut chip thickness being approximately micro size compared to the circular radius of the cutting edge. Thus, the length of the tertiary and the quaternary zones is equal to the primary shear zone, which is proposed to be the arc length of contact part *L*. 

(3) $$\begin{array}{l} L=AB+BC+CD=\theta_{m}\frac{1}{180}\pi r_{e}+\alpha_{0}\frac{1}{180}\pi r_{e}+\frac{\Delta t-r_{e}(1-\cos\alpha_{0})}{\sin\alpha_{0}}\\ =\frac{1}{180}\pi r_{e}(\arccos(\frac{r_{e}-t_{\min}}{r_{e}})+\alpha_{0})+\frac{\Delta t-r_{e}(1-\cos\alpha_{0})}{\sin\alpha_{0}} \end{array}$$

Due to the high speed and the large deformation of the material in the micro-milling process, the size effect on the cutting process is considered in the third and the fourth regions between the cutter round edge and the work material. The work material is considered as a plastically isotropic material. In order to analyze the size effect, the strain gradient plasticity is introduced in micro machining. 

Taylor’s dislocation model based on the strain gradient plasticity gives the flow stress in terms of the dislocation density as:

(4) $$\sigma =\sqrt{3}\alpha_{c}Gb\sqrt{\rho_{total}}=\sqrt{3}\alpha_{c}Gb\sqrt{\rho_{ssd}+\rho_{gnd}}$$

where *α*_c_ is a constant value to be taken from 0.3 to 0.5, *G* is the material shear modulus with a unit of MPa, *b* is the magnitude of Burgers vector with a unit of nanometer. The total dislocation density *ρ*_total_ is given by the sum of the density of statistically stored dislocations *ρ*_ssd_ and geometrically necessary dislocations *ρ*_gnd_.

The density of statistically stored dislocations *ρ*_ssd_ can be determined by the material test based on the uniaxial stress–strain law in the absence of strain gradient effect as:

(5) $$\sigma =\sqrt{3}\alpha_{c}Gb\sqrt{\rho_{ssd}}=\sigma_{c}=\sigma_{JC}$$

where *σ*_c_ is the conventional flow stress of work material in macro machining, which can also be obtained from the Johnson–Cook model [27]. The *σ*_JC_ is the material tensile reference stress in the uniaxial tension with a unit of MPa.

The density of geometrically necessary dislocations *ρ*_gnd_ is given by the effective strain gradient *η* as:

(6) $$\rho_{gnd}=\frac{\bar{r}\eta }{b}$$

where $\bar{r}$ is Nye coefficient to express the distribution of GNDs corresponding to the effect of crystallography, which is about two for the polycrystalline material [28].

The value of strength coefficient *μ* is necessarily introduced for estimating the total dislocation density, which is given as 0.38 [2] in micro machining.

Substituting Equations (5) and (6) into Equation (4), the flow stress can be written as:

(7) $$\sigma =\sigma_{JC}\sqrt{1+{\left(\frac{\rho_{gnd}}{\rho_{ssd}}\right)}^{\mu }}=\sigma_{JC}\sqrt{1+{\left(\frac{3\bar{r}\eta \alpha_{c}{}^{2}G^{2}b}{\sigma_{JC}{}^{2}}\right)}^{\mu }}$$

The strain gradient based on the Joshi [29] model is obtained by the analysis of the density of geometrically necessary of shear zone for micro scale machining, as follows:

(8) $$\rho_{gnd}=\frac{1}{bL}$$

Based on Equations (6) and (8), the strain gradient is rearranged as:

(9) $$\eta =\frac{1}{\bar{r}L}$$

Thus, a flow stress equation can be given as:

(10) $$\sigma =\sigma_{JC}\sqrt{1+{\left(\frac{3\alpha_{c}{}^{2}G^{2}b}{\sigma_{JC}{}^{2}L}\right)}^{\mu }}$$

A flow stress is expressed according to model of Subbiah [30], which is based on ductile fracture theory. 

(11) $$\sigma_{fracture}=\frac{2(1-\nu )K^{2}}{\pi Gb}$$

The *σ*_fracture_ is the uniaxial flow stress deduced by fracture mechanics when the material is about to break, *ν* is the Poisson ratio of material, and *K* is the value of fracture toughness, which is stress intensity factor.

Flow stress in the tertiary and the quaternary zones is comparable to fracture toughness when the uncut chip thickness is equal to the minimum chip thickness. Hence:

(12) $$\sigma_{fracture}=\sigma$$

Substituting Equation (3) into Equation (10) and simultaneously using Equations (1), (9), and (11), the new minimum chip thickness is derived as follows:

(13) $$\begin{array}{l} t_{\min}^{\prime }=r_{e}(1-\cos\gamma ) \end{array}$$

wherein, $\gamma =\frac{3\alpha_{c}{}^{2}G^{2}b}{{\left[{\left(\frac{2(1-\nu )K^{2}}{\pi Gb\sigma_{JC}}\right)}^{2}-1\right]}^{\frac{1}{\mu }}\sigma_{JC}}\frac{180}{\pi r_{e}}-\alpha_{0}-\frac{\Delta t-r_{e}(1-\cos\alpha_{0})}{\sin}\alpha_{0}\frac{180}{\pi r_{e}}$.

From the aforementioned deduction and analysis for formulations, the proposed minimum chip thickness model can reflect the characteristics of micro machining in the third and the fourth material areas, including the material strengthening behaviors and the friction relationship between the material and the cutting edge.

Thus, the minimum uncut chip thickness is not only dependent on the arc radius of the cutting edge but also on properties of the work material in tertiary and quaternary zones.

### 2.2. Micro Machined Surface Roughness

**Figure 2:**
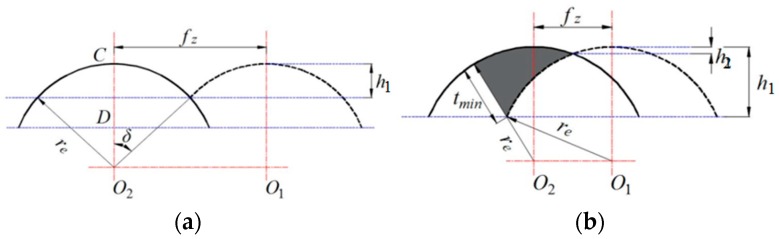
Plane of ideal feed trace of micro groove bottom surface. (**a**) The cutting edge radius is smaller than the feed rate per tooth. (**b**) The cutting edge radius is larger than the feed rate per tooth.

The small part of the material is not cut off and is therefore left on the machined surface in the microscale milling due to the relative motion relationship between cutter and work material, upon which the height of the residual area has direct influence on the machined surface roughness [17,26,31]. The theoretical residual area refers to the micro uneven degree of the machined surface. The arithmetic average roughness *R*_a_ is, in practice, generally used to characterize the surface flatness *h* of the machined surface. The *R*_a_ is theoretically 4~5 times that of *h*. The surface roughness is actually much higher than the theoretical residual area, in which height *h* can be calculated according to the geometric relationship, as shown in Figure 2 [12].

When the radial edge of the cutter is smaller than the feed rate per tooth, the height *h*_1_ can be obtained by the geometric relationship, as shown in Figure 2b. The following equation can be expressed as:

(14) h1=O2C−O2D=re−re2−(fz2)2=re−re2−(30vfNn)2=re(1−1−sin2δ)≈resin2δ22≥fz28re

where *r*_e_ is the radius of the cutter edge, *f*_z_ is the feed rate per tooth, *v_f_* is the feed rate of the cutter, *N* is the number of cutter flutes around the micro cutters, *n* is the spindle speed, and *δ* is the overlapping angle for the edge radius.

The flatness of the groove bottom is mainly determined by the feed rate per tooth and the spindle speed of the cutter without considering wear of micro cutter edge, as can be seen from Equation (14) when the cutting edge radius is smaller than the feed rate per tooth. The greater the feed rate per tooth *f*_z_ is, the greater the surface roughness value will be. The high spindle speed is in contrast to the feed rate per tooth, which leads to lower surface roughness. At a constant feed rate per tooth, by increasing the radius of the cutter edge (considering the wear of the cutter), the surface roughness value decreases. 

When the radius edge of the cutter is greater than the feed rate per tooth, the height *h*_2_ takes the minimum cutting thickness *t’*_min_ into account, which can be obtained by the geometrical relationship, as shown in Figure 2b. The following equation can be expressed as:

(15) $$h_{2}=\frac{f_{z}{}^{2}}{8r_{e}}+\frac{t_{\min}^{\prime }}{2}\left(1+\frac{r_{e}t_{\min}^{\prime }}{2}\right)$$

The theoretical value of flatness can be changed with increase or decrease of the feed per tooth and the minimum chip thickness without wear of the cutter edge radius. 

Combining Equations (13) and (15) yields another form of the equation of the height *h*_2_.

(16) $$h_{2}=\frac{f_{z}{}^{2}}{8r_{e}}+T\left(1+r_{e}T\right)=\frac{f_{z}{}^{2}}{8r_{e}}+T^{2}\left(\frac{1}{T}+r_{e}\right)$$

in which:

(17) $$T=\frac{r_{e}}{2}(1-cos\gamma )$$

The minimum chip thickness is also determined by the instantaneous undeformed chip thickness during cutter rotation. The instantaneous undeformed chip thickness *t*(*θ*) without run out and tilt of the micro cutter can be approximately given by the following Equation (18) [32]:

(18) $$t\left(\theta \right)=R\left[1-\sqrt{1-\frac{2f_{z}\sin\theta }{R+\frac{Nf_{z}}{2\pi }\cos\theta }-\frac{f_{z}{}^{2}\cos(2\theta )}{{\left(R+\frac{Nf_{z}}{2\pi }\cos\theta \right)}^{2}}+\frac{f_{z}{}^{3}\sin\theta \cos^{2}\theta }{{\left(R+\frac{Nf_{z}}{2\pi }\cos\theta \right)}^{3}}}\right]$$

where *θ* is the angular position of the micro tooth, *R* is the radius of the micro edge cutter, *f*_z_ is the feed rate per tooth, and *N* is the number of cutting flutes around the micro cutters. Thus, the instantaneous angular angle has a large impact on the determination of minimum chip thickness.

The micro groove surface topography model previous is described under theoretical circumstances. In reality, the cutting process is a trochoid trajectory of the cutting edge. The position of the *i*th flutes of the cutter edge tip without considering cutter run out in the Cartesian coordinate system *XOY* can be represented by:

(19) $$\left\{ \begin{array}{l} x_{i}=\frac{f_{z}N}{2\pi }\theta +R\sin(\theta +\frac{2\pi (i-1)}{N}+\theta_{0})\\ y_{i}=R\cos(\theta +\frac{2\pi (i-1)}{N}+\theta_{0}) \end{array}\right.$$

where *θ*_0_ is the initial position angle of the cutter edge tip in the coordinate system. It is assumed that the initial position of the cutter edge tip is located at a positive direction of the *Y* axis. Some of the cutting parameters are selected as *f*_z_ = 0.2 μm/z, *N* = 2, *R* = 10 μm. The workpiece material moves along the negative cutting direction of the *X* axial, and the micro cutter rotates in a clockwise cutting direction.

**Figure 3:**
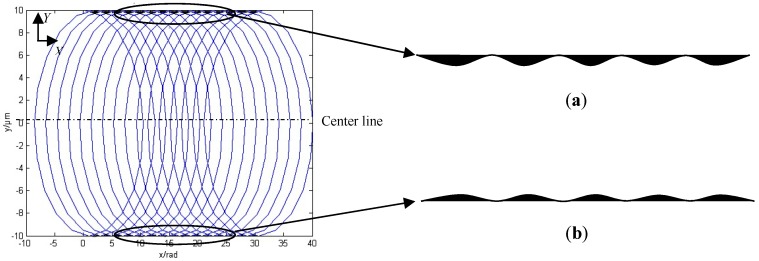
The micro groove surface topography without considering total radial run out. (**a**) Up milling. (**b**) Down milling.

By substituting the cutting parameters and the previous assumption into Equation (19), the micro groove surface topography is obtained, as shown in Figure 3.

At both sides of the micro groove wall exists a certain cutting dead area that refers to residual material not cutting. It is found from an amplification program in Figure 3 that the height of the residual material zone is slightly larger in up milling than in down milling. The chip thickness on the bottom of the groove is of uniform distribution, and there is no machining blind zone in theory.

**Figure 4:**
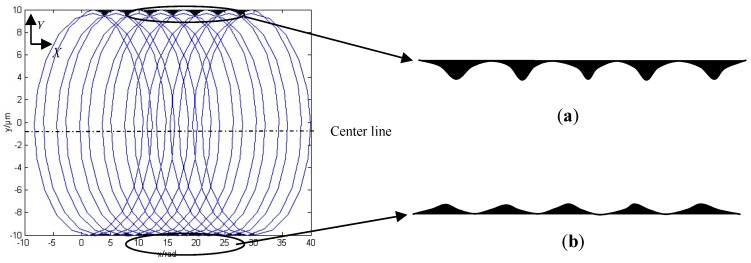
The micro groove surface topography considering total radial run out. (**a**) Up milling. (**b**) Down milling.

The position of the *i*th flutes of the cutter edge tip considering cutter run out in the Cartesian coordinate system *XOY* can be expressed as:

(20) $$\left\{ \begin{array}{l} x_{i}=\frac{f_{z}N}{2\pi }\theta +R\sin(\theta +\frac{2\pi (i-1)}{N}+\theta_{0})+r_{0}\sin(\theta +\psi )\\ y_{i}=R\cos(\theta +\frac{2\pi (i-1)}{N}+\theta_{0})+r_{0}\cos(\theta +\psi ) \end{array}\right.$$

where *r*_0_ is the total radial run out of the cutting edge, including the spindle run out and the tool manufacturing and assembling errors, and *ψ* is the tool run out angle. Thus, on the basis of the previous data, the total radial run out is 0.3 μm, and the tool run out angle is 0.005π. The micro groove surface topography is shown in Figure 4.

**Figure 5:**
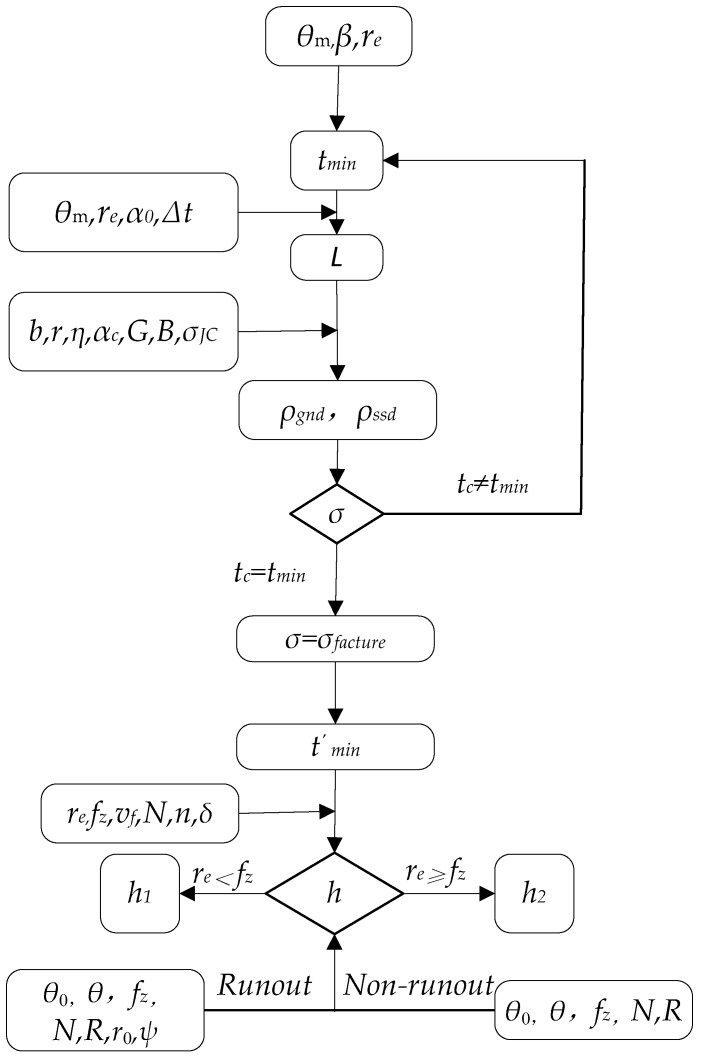
Flow chart of the surface model.

The micro groove surface topography is largely different from that without tool run out. It is obviously seen that the height and the width of the cutting dead zones are greater than those without considering run out. It is also found from the amplification program in Figure 4 that the height of the cutting blind zone is significantly higher in up milling than in down milling. The widths of the trajectories are of non-uniform distribution on the bottom of the micro groove. Thus, the surface quality of the micro groove could deteriorate further with an increase of the total radial run out. Furthermore, the width and the height with respect to the cutting dead zone of the micro groove are larger between the final residual material and the minimum chip thickness, considering the minimum chip thickness Equations (1) and (13), the instantaneous chip thickness Equation (18), the material properties, and so on. According to the surface formation model and the surface roughness model above, the flow chart of the surface model is shown in Figure 5.

In order to further investigate the aforementioned proposed model, several experimental cuttings were performed and are presented as follows. 

## 3. Experimental Details

### 3.1. Setup for Experiments

**Figure 6:**
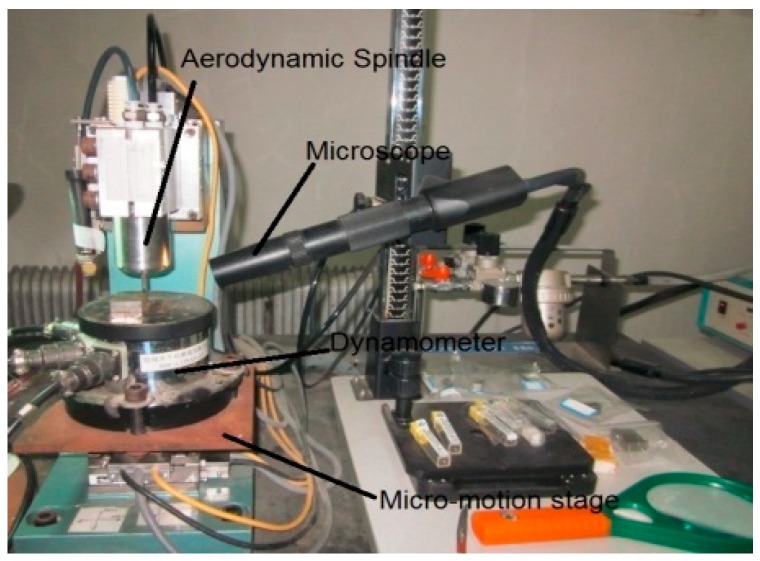
The micro-milling experiment.

**Figure 7:**
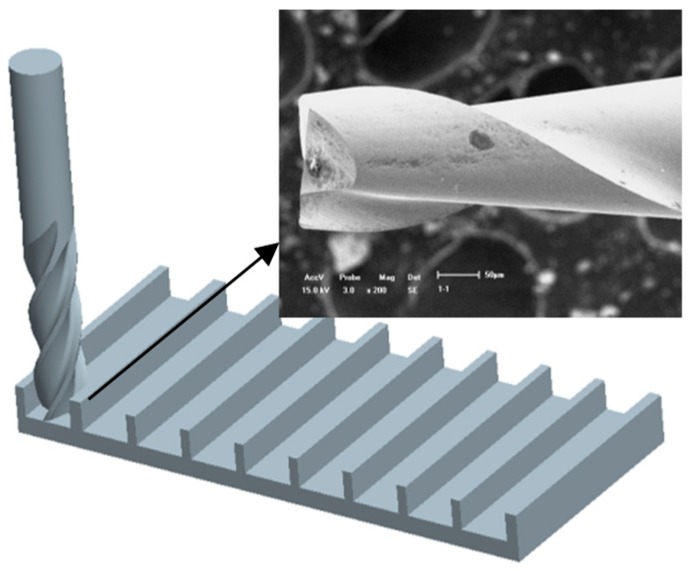
Micro groove milling sketch view.

A series of milling tests under dry cutting conditions were performed on the three-axis micro-machining center, as shown in Figure 6. A two-flute and uncoated micro-end milling cutter made of the Cemented Carbide (WC) with a shank diameter of 3.175 mm and a blade diameter of 0.3 mm was utilized for several of the material blocks (20 mm × 10 mm × 3 mm) in the experiments. The feed motion of the machine platform was driven by the MX80L system. The limit value of the stage was 50 mm, the detecting resolution was 10 nm, and the repeatability and the uncertainty were found to be less than 0.1 μm. Precise closed-loop and multi-axis control of each micro motion platform was driven by a 6 K motion controller and the Vix linear driver, in which the range of the feed speed was 0.001 mm/s~10 mm/s. The spindle system adopted air turbine with a maximum rotational speed of 1.6 × 10^5^ rpm, and the rotational accuracy was about 1 μm. The micro tool run out was measured to be 0.3 μm. The series of feed rates, spindle speeds, and axial cutting depths were determined in tests. After every preceding specimen block was cut, the micro cutter was cleaned to avoid built-up edge, which would have affected the surface roughness of the following cutting experiments. The micro edge of the tooth was also checked to make sure that it was properly sharp [14]. The micro-milling grooves sketch map is shown in Figure 7.

### 3.2. Materials Behaviors

In order to investigate the relationship between surface roughness and material properties, the six selected materials in this test consisted of the aluminum alloys 5083-O and 6082-T6, the titanium alloy Ti6Al4V, the 45 steel, the stainless steel 304, and the tool steel SKH-9. The mechanical properties and the parameters of the materials are summarized in Table 1.

### 3.3. Approach Design 

The orthogonal test table L_25_(5^3^) was designed to study the effect of the spindle speed, the feed speed, and the axial depth and how they respectively corresponded to the surface roughness. Factor level codes and values of cutting parameters are listed in Table 2 and Table 3.

A series of straight slots with a length of 10 mm and a width of 0.3 mm were milled at a time according to the parameters of Table 2 and Table 3. In order to quantitatively measure the surface of the grooves, the surface topology was measured under SEM and the Keyence VHX-10003D Super Depth Digital Microscope (SDDM). The surface roughness ranged from 0.2 mm × 0.2 mm, corresponding to the micro grooves measured by the STIL 3D Laser Profiler Measuring Apparatus, in which the minimum resolution was 0.1 nm. The repeatability and the uncertainty of the measurements were ±0.01 nm and±0.03 nm, respectively. Ten different regions of the same micro groove were repeatedly measured, and the mean of the ten measurements was taken as the experimental result to make the experimental results more realistic and reliable.

## 4. Experimental Results

**Figure 8:**
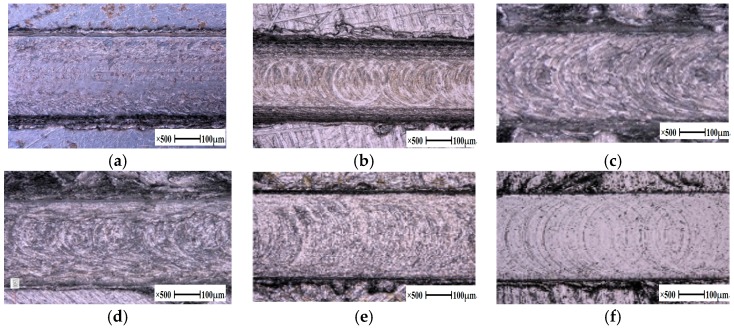
High view microscope picture of micro grooves versus material. (**a**) 45, (**b**) 304, (**c**) 5083-O, (**d**) 6082-T6, (**e**) SKH-9, (**f**) Ti6Al4V.

The micro grooves corresponding to six different materials were cut according to setting parameters, which were all previously established. The surface topographies of the grooves were clearly observed under SEM and measured under SDDM, as shown in Figure 8. 

Because the titanium alloy Ti6Al4V is so widely used in all fields, larger experimental data and detailed analysis are only listed for this alloy herein. The analysis method of the other selected materials is the same as that of alloy Ti6Al4V. 

**Figure 9:**
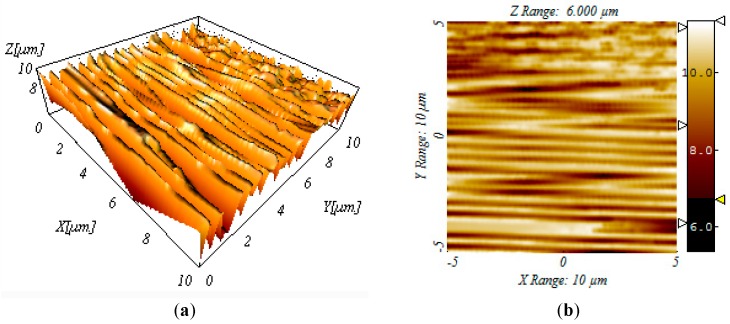
Groove bottom surface topography for Ti6Al4V. (**a**) Surface 3D topography. (**b**) Surface 2D topography.

In order to quantitatively measure the micro groove change to obtain the surface roughness values, the groove bottom surface topography was measured using the STIL 3D Laser Profiler Measuring Apparatus. Figure 9 shows an example of measured surface topology and extracted surface profile. It is clear that there were obvious processing textures on the machined surface of the workpiece along the feed direction. The material on both sides of the groove was stretched, and the cutting marks showed obvious plastic deformation and ploughing/rubbing. Table 4 shows the mean of the ten measurements at five different regions for the same zone (200 μm × 200 μm) of the micro grooves with respect to surface roughness and range analysis of Ti6Al4V.

The *R_j_* in Table 4 reflects the amplitude of variation with respect to the test index when the level of the j-th factor fluctuated. The greater the *R_j_* was, the greater the effect of the factor on the test index was. It can be judged that the feed rate was the main factor affecting surface roughness due to *R_f_*_s_ > *R*_n_ > *R_a_*_p_, following spindle speed and axial depth, respectively.

**Figure 10:**
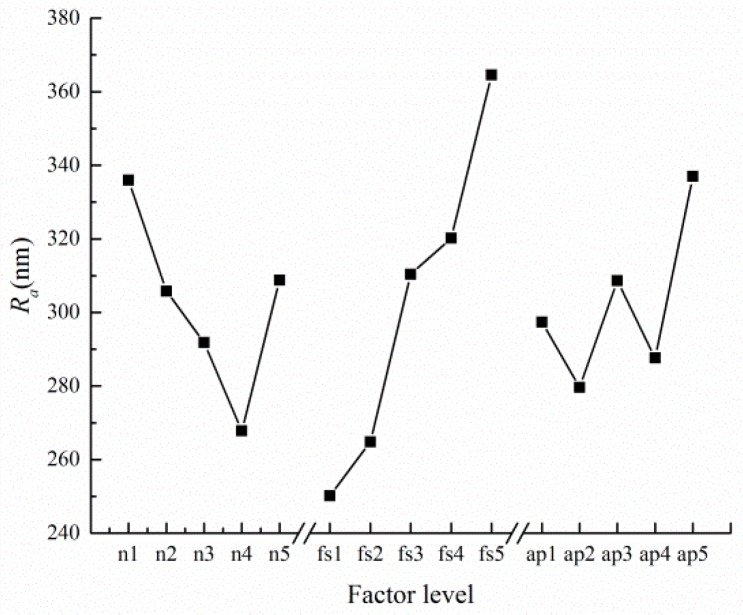
Factor and surface roughness tendency chart.

In order to further investigate the influence of the factors with experimental indicators in reference to cutting parameters, the tendency chart of factor level versus surface roughness is plotted in Figure 10. It can be more intuitive to reflect the surface roughness changes with factor level. The level of factors can be selected for further testing.

## 5. Discussion and Analysis

In this section, the theoretical model with respect to the micro groove surface is examined with experimental validation to further investigate the forming mechanism of the metal micro surface for six different materials. The relationship of the surface roughness and the processing parameters is plotted according to the experimental data measured in Table 4, as shown in Figure 9.

### 5.1. The Cutting Speed for the Surface Roughness

**Figure 11:**
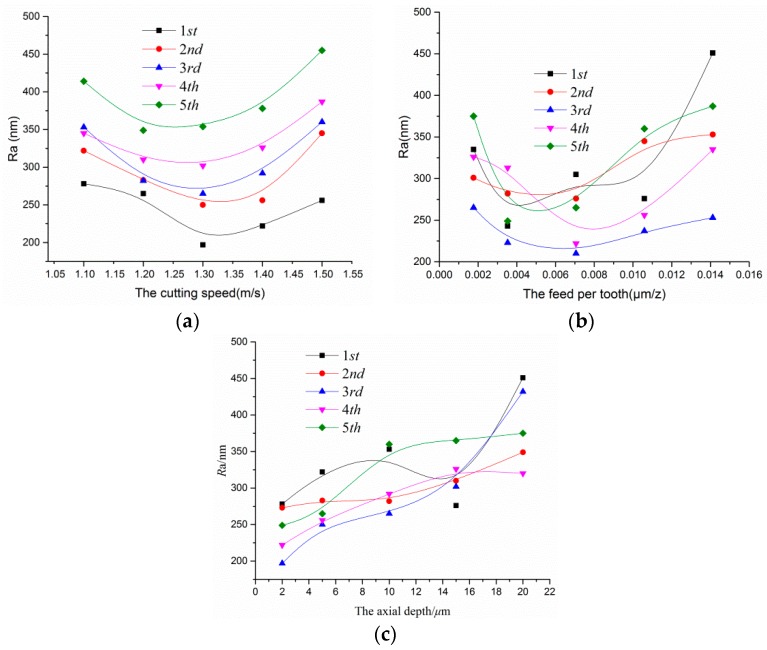
Effects of processing parameters on *R_a_* for Ti6Al4V. (**a**) Change in *R_a_* with the cutting speed. (**b**) Change in *R_a_* with the feed per tooth. (**c**) Change in *R_a_* with the axial cutting depth.

The surface roughness (*R*a) decreased remarkably with the increase of the cutting speed when the spindle speed was approximately less than 1.3–1.35 m/s. The minimum value the Ra reached was at about 1.32m/s. This trend had opposite results compared to those of the macro-scale curve, as shown in Figure 11a.

There was a sudden change in the vicinity of the critical value. That is to say, the surface roughness of the micro groove was related to the temperature or the force generated between the material and the micro cutter edge in the micro-milling process.

The spindle speed at 82,500–85,000 rpm was the critical cutting speed according to macro-scale theory, i.e., the cutting linear speed was 1.25–1.35 m/s.

The undeformed chip layer was bifurcated upward into the first and the second deformation zones and downward into the third and the fourth deformation zones when the cutting speed was less than 1.32 m/s. Part of the material ran along the rake face and formed a chip, and the rest of the material, through extruding and scratching, generated the finished, machined surface, as shown in Figure 1.

As the cutting speed lowered and the chip flow slowed, a large amount of heat was produced due to higher friction accumulated between the micro cutter edge and the cutting zone. At the same time, the higher friction caused a downward flow of material or plough when the instantaneous cutting depth was below the minimum cutting thickness at a lower speed. Thus, most of the energy was absorbed by the machined surface and the cutter edge, and the temperature during the cutting process continued to rise and soften the material.

When the cutting speed was much than 1.4 m/s, the faster the chip flow, the better the machined surface quality, which attributes to the 70–80% of the cutting heat was taken away [34,35].

The friction between the first and the second deformation zones and the cutter edge experienced further increase, and the frequency for the material in the third and the fourth zones was scratched and compressed, which increased and intensified the cutter edge wear. The cutter edge radius also became large, as shown in Figure 1. In the case of uncut chip thickness remaining unchanged, as the stagnant angle and the frictional angle increased (as in Equations (1) and (13)), the minimum chip thickness increased. Consequently, the cut chip thickness actually decreased, and the amount of chip reduced while the chip flow became larger.

As the stagnation point A moved upward along the micro cutter edge, most of the material in the first and the second deformation zones flowed into the third and the fourth deformation zones. In addition, there was higher speed, larger eccentricity of the micro tool, and greater impact between the tool and the surface being machined, which resulted in a large number of scratches and worse surface morphology. The magnitude of surface roughness correspondingly increased.

### 5.2. The Feed Per Tooth for the Surface Roughness

According to the cutting speed in Figure 11a, the lower surface roughness corresponded to1.3 mm/s, thus it was used for the feed per tooth. By increasing the feed per tooth of the micro cutter, the value of surface roughness of the corresponding micro grooves being machined decreased. Then, the value gradually increased as the feed per tooth became greater than certain values or ranges, as shown in Figure 11b. According to the literature [36], the larger the feed rate is, the larger the amount of feed per tooth will be when the spindle speed is a constant value in the micro-milling process. Figure 3 shows that the trochiodal trajectories for the bottom of the micro grooves were of ideally uniform distribution, and the intervals between the trochiodal trajectories became larger as the feed per tooth increased.

The material being cut was only extruding and scratched when the feed per tooth was less than the minimum chip thickness and could be not removed. Local materials continued to accumulate in front of the micro cutter edge with the rotation of the spindle sustained. Once the breaking strength was exceeded with respect to the material, plastic deformation occurred when the chip thickness reached the minimum chip thickness, and the chip was generated and removed.

Due to the feed per tooth being very small in the high speed spindle, the material being machined in the vicinity of the cutter edge formed a chip that could not be removed during the first rotation of the spindle. With a last, increasing revolution of the spindle, the certain chip thickness was removed. With the help of the finite element numerical simulation and a high speed camera, the phenomena could be observed. The trend of sliding friction and extrusion was obviously much from the center line. The details about the value of surface roughness in the micro grooves can be found in the research of Gong [16].

The feed speed was less than approximately 0.004–0.006 μm/z, which was less than the minimum chip thickness. Increasing the feed per tooth caused the values of surface roughness to decrease, as shown in Figure 11b. This could be attributed to the machined material only sliding and extruding and the fact that no material was removed. Similarly, if the surface being machined was completely rolled, the surface quality would be improved.

Figure 11b shows values of surface roughness, and it is interesting to note that, as the feed per tooth increased to around 0.007–0.008 μm/z, there was a gradual but noticeable increase as the feed speed further increased. This could have been because the material being machined near the center line was sheared, and further away, it was gradually being rubbed and squeezed, as shown in Figure 3 and Figure 4. As the feed speed increased in the micro cutter, the ploughed trend occurred in both grooves more obviously. The integrity of the surface relative to the whole area being measured declined. The trend of the latter is in good agreement with the traditional theory [1]. This could be obtained and calculated by Equations (14)–(16). The maximum variation of roughness was about 120 nm when the feed speed was less than 0.003 μm/z, while the roughness value varied greatly when the feed speed was greater than 0.004 μm/z.

### 5.3. The Axial Cutting Depth for the Surface Roughness

With the depth increasing in the axial cutting, the values of surface roughness increased in a wave-like way, which is shown in Figure 11c. This was because the increased cutting depth caused the cutting area, the force, and the radial deformation to increase as well, which resulted in unusual cutting and an increase of surface roughness.

The surface roughness in the third experiment was negative, which may have been caused by the minimum cutting thickness effect and the strain gradient plasticity.

According to the literature [28], the cutting depth is generally a few tens of microns in the high speed milling process, and the force fluctuation is smaller, i.e., the variation of surface roughness is lower in the steady-state milling. If the cutting depth increases, the force greatly rises, which results in deformation of the workpiece and tool vibration. The surface roughness varies obviously. However, too small a cutting depth also causes ploughing, scratches, as well as shortened life of the tool. Thus, by taking into consideration the influence of various factors affecting the surface roughness with respect to the cutting depth, a reasonable value is identified.

### 5.4. The Structural Material for the Surface Roughness

Under the same conditions of processing, the microstructures of the micro grooves of each material are shown in Figure 8.

Figure 8 displays the different trochoid trajectories that remained on the slot surface were in good agreement with the theoretical analysis in Section 2. 

It was obvious that there were varying degrees of scratches on both sides of the micro slots, and some of those had a few pits due to fracture or spalling. 

**Figure 12:**
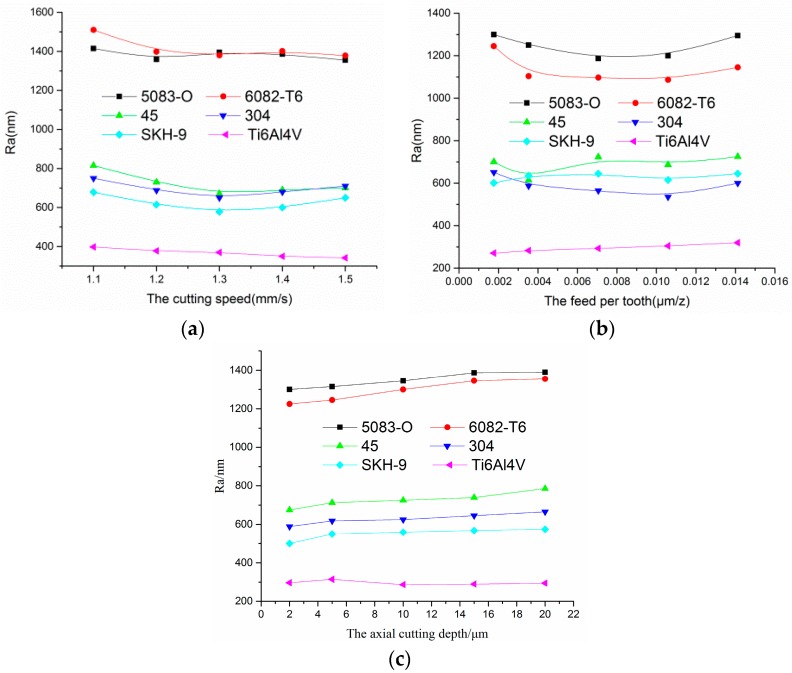
Influence of milling parameters on surface roughness of different materials. (**a**) Change curves of *R*_a_ with the cutting speed (*f*_s_ = 20 μm/s, *a*_p_ = 10 μm). (**b**) Change curves of *R*_a_ with the feed per tooth (*n* = 83,200 rpm, *a*_p_ = 10 μm). (**c**) Change curves of *R*_a_ with the axial cutting depth (*n* = 83,200 rpm, *f*_s_ = 20 μm/s).

The trajectories and the scratches corresponding to each material were greatly distinguished from one another. With the aim of revealing the existing relationship between surface roughness and the mechanical properties of materials as well as the processing parameters of milling, the surface roughness relative to the selected materials is given in Figure 12.

It is clearly seen that the variation trends of surface roughness to the same kinds of material were identical. The magnitudes for surface roughness of materials based on their values could be approximately divided into three categories. Firstly, the amplitudes of surface roughness for the aluminum alloys 5083-O and 6082-T6 were in close range, from approximately 1.250–1.580 μm. In the second category, the variation magnitudes of the 45 steel, the stainless steel 304, and the tool steel SKH-9 had a range of approximately 0.4–1 μm. Finally, the Ti6Al4V was obviously located in the last category, as the value of roughness was less than 0.4 μm.

Considering the results from the representation above, it should be possible to account for the influence of material properties, including physics and mechanics, as shown in Table 1. 

From a physical point of view, the thermal conductivity of titanium alloy Ti-6Al-4V was the smallest, while those of aluminum alloys 5083-O and 6082-T6 were the largest.

The heat was transferred out less during processing with smaller thermal conductivity, and it was largely accumulated in the vicinity of the micro edge, which resulted in higher energy in the micro cutting zone, making the materials softer and easier to cut. Under conditions of the same processing parameters, for example, the cutting temperature for the Ti-6Al-4V was higher than that of the aluminum alloy even though Ti-6Al-4V is harder, stronger, and less ductile than the aluminum alloy at room temperature. This is because the thermal softening effect made Ti-6Al-4V more ductile at the corresponding cutting temperatures compared to aluminum alloy at those same temperatures. Also, according to literature [6], the thermal softening effect and the strain hardening effect are equally important and cancel each other out when micromachining Al6082-T6. Thus, the Ti-6Al-4V was easier to cut than the 5083-O and the 6082-T6, the value of surface roughness was smaller, and the quality of the machined surface was better than others.

From a mechanical point of view, due to the machined materials all having plastic behavior properties, the tensile strength and the hardness of the aluminum alloy under the same conditions were smaller than 45 steel, 304, SHK-9, and Ti-6Al-4V. The materials with much higher plastic behaviors reached the fracture stage under the action of milling first, while those with lower plastic behaviors lagged behind. Thus, the latter scenario greatly decreased the extent of ploughing and scratching in the cutting processing. Therefore, the values of surface roughness corresponding to 45 steel, 304, and SHK-9 were lower. 

In addition, the elongations of 45 steel and stainless steel 304 were larger than that of SKH-9, which lead to bigger plastic deformation, strong hardening coefficient, and more contact area with the micro cutter edge. In view of the hardening coefficient in particular, it is easy to produce a built-up edge and burrs that have a direct influence on the surface being machined. 

### 5.5. The Run Out for the Surface Roughness

Burrs on both side walls of the micro grooves of the aluminum alloy had a continuous distribution, and their size was large relatively, as shown in Figure 8c,d. The burrs were relatively uniform, while their size was small, corresponding to 45 steel and SKH-9, as presented in Figure 8a,e. Burrs on both side walls were rare, but the width of the micro groove was smaller than the actual size for stainless steel 304, as illustrated in Figure 8b. There were almost no burrs on the sides, while the width of the micro groove was in agreement with the actual size of the titanium alloy, as shown in Figure 8f.

**Figure 13:**
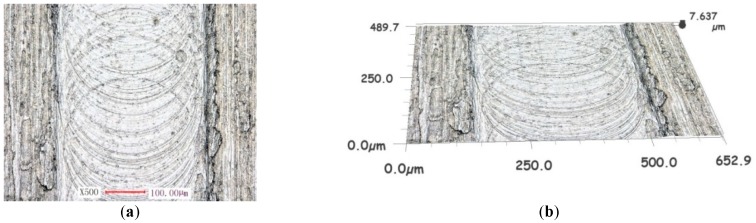
The micro groove surface topography for Ti-6Al-4V without run out. (**a**) Surface of micro groove. (**b**) Topography of micro groove.

**Figure 14:**
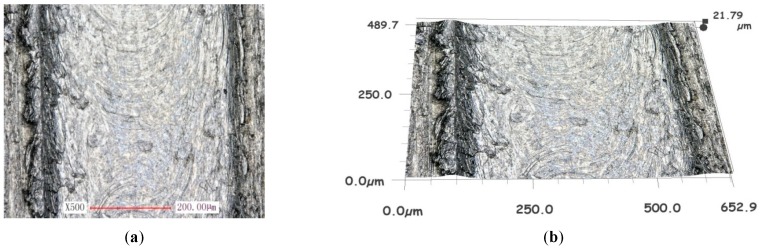
The micro groove surface topography for Ti-6Al-4V with run out. (**a**) Surface of micro groove. (**b**) Topography of micro groove.

The generation and the formation of the burr involved a great number of factors, such as cutter wear, material properties, tool run out, processing parameters, etc. The effect of the micro tool run out with respect to the titanium alloy was analyzed and is presented in Figure 13 and Figure 14.

Under the conditions of the same cutting processing parameters, the maximum height of the burrs on both side walls was measured to be 7.637 μm without run out, as shown in Figure 13b, while the maximum height with a value of run out equal to 0.3 μm was measured to be about 21.79 μm, as presented in Figure 14b.

The burrs in the up milling zone were greater than those in the down milling area of the micro groove. With the quantity level increasing the run out amplitudes, the residual burrs became larger.

The experimental results presented above are in good agreement with the theoretical analysis in Section 2.2. 

It can be seen in Figure 13 and Figure 14 that the width of the micro groove was larger than the actual diameter of the micro cutter by around 300 μm, which could not be analyzed by the theory in Section 2.2. It could be attributed to the fact that, under conditions of micro edge radius not being worn, the principal cause of slot enlargement was radial run out of the micro cutter.

The surfaces being machined had poor quality, and the micro cutter edges were seriously worn, thus some of them may have fractured, for example, due to the run out of the micro cutter, which made it possible for the cutting to not engage in the materials [36,37,38].

Compared with a conventional cutter, the micro cutter with characteristics of smaller diameter, higher spindle speed, poorer rigidity, and alternating load had a prominent run out.

The dynamic instability was strengthened when the run out was comparable in size to the feed per tooth. For the cutter with two teeth, especially, it was easy to form cutting on only a single tooth.

The vibration of the machine tool, the imbalance of the spindle rotation, the asymmetry of cutter geometry, the periodic change of cutting force, etc., could cause the radial run out of the cutter in the micro machining process, which would result in widening the width of the grooves being machined.

The cutter is not perpendicular to the surface being machined and does not engage in the material if the ratio of radial run out to cutter diameter in micro machining is more than ten times (or tens of times) that of conventional milling. 

Under the previous conditions, self-excited vibration occurred due to the dynamics variation of the chip thickness. In such situations, the surface quality of the micro grooves may deteriorate further in the processing of micro-machining [9].

## 6. Conclusions

The following conclusions can be drawn from this paper.

The surface formation model was developed based on the strain gradient plasticity of materials for the prediction of the minimum chip thickness. The model predicts the minimum chip thickness from properties of the work material and the friction relationship between the material and the cutting edge.

The micro surface roughness model considering scenarios both with and without run out of the cutter was proposed for the prediction of surface quality corresponding to micro grooves.

The model was used to justify experimental results over the orthogonal test using a different variety of materials as workpieces.

The feed rate and the spindle speed in the machined parameters have significant effects on the surface roughness. In addition, the physical and mechanical properties of the material being machined must be considered, as it should be possible to account for the extent to which the micro radius and the cutting edges of the cutter influence the strain gradient effect. The surface of the titanium alloy Ti6Al4V was found to stay at lower values over a range of cutting conditions. Compared to other materials, this was attributed to the predominance of the thermal softening effect over the minimum uncut chip thickness effect.

The model—both considering and not considering cutter run out—was applied for the prediction of micro groove surface. This model was also experimentally validated over a range with and without run out cutting conditions. The smaller the run out is, the better the surface quality will be. 

## Figures and Tables

**Table 1:** Mechanical properties of the workpiece [33].

Materials	*ρ* [kg/m^3^] × 10^−3^	*HB* [N/m^2^] × 10^−4^	*σ*_b_ [Pa] × 10^9^	*δ*_5_ [%] × 10^2^	*E* [Pa] × 10^11^	*K* [W/m·k] × 10
5083-O	2.70	0.852	≥0.270	≥0.12	0.720	8.876
6082-T6	2.70	1.05	≥0.205	≥0.14	0.725	8.878
Ti6Al4V	4.40	1.95	0.539	≥0.10	1.100	0.795
45	7.85	2.29	≤0.355	≥0.16	2.100	4.768
304	7.93	≤1.87	≥0.520	≥0.40	1.985	1.540
SKH-9	8.61	≤2.62	3.430	–	2.180	1.675

**Table 2:** Slot milling factor level code.

Factor	*n* [rpm] × 10^4^	*f*_s_ [μm/s]	*a*_p_ [μm]
1	7.04	5	2
2	7.68	10	5
3	8.32	20	10
4	8.96	30	15
5	9.60	40	20

**Table 3:** Comparison of cutting speed and spindle speed.

*n* (rpm)	70,400	76,800	83,200	89,600	96,000
*v* (m/s)	1.1	1.2	1.3	1.4	1.5

**Table 4:** Experimental measurements for Ti6Al4V.

N	*n*/rpm	*f*_s_/μm/s	*a*_p_/μm	*f*_z_/10^−3^ μm/z	*R*_a_/nm
1	70,400	5	2	2.13	278
2	70,400	10	5	4.26	322
3	70,400	20	10	8.52	353
4	70,400	30	15	12.8	276
5	70,400	40	20	17.1	451
6	76,800	5	20	1.95	305
7	76,800	10	2	3.91	283
8	76,800	20	5	7.81	282
9	76,800	30	10	11.7	310
10	76,800	40	15	15.6	349
11	83,200	5	15	1.80	197
12	83,200	10	20	3.60	250
13	83,200	20	2	7.21	265
14	83,200	30	5	10.8	302
15	83,200	40	10	14.4	445
16	89,600	5	10	1.67	222
17	89,600	10	15	3.35	256
18	89,600	20	20	6.70	292
19	89,600	30	2	10.0	326
20	89,600	40	5	13.4	243
21	96,000	5	5	1.56	249
22	96,000	10	10	3.13	213
23	96,000	20	15	6.25	360
24	96,000	30	20	9.38	387
25	96,000	40	2	12.5	335
*K*_1_	1680	1251	1487		
*K*_2_	1529	1324	1398		
*K*_3_	1459	1552	1543		
*K*_4_	1339	1601	1438		
*K*_5_	1544	1823	1685		
$\bar{K_{1}}$	336	250.2	297.4		
$\bar{K_{2}}$	305.8	264.8	279.6		
$\bar{K_{3}}$	291.8	310.4	308.6		
$\bar{K_{4}}$	267.8	320.2	287.6		
$\bar{K_{5}}$	308.8	364.6	337		
*R*_j_	68.2	114.4	57.4		
Sequence	*f_s_ > n > a_p_*				
